# *In Vivo* and *In Vitro* Studies Suggest a Possible Involvement of HPV Infection in the Early Stage of Breast Carcinogenesis via APOBEC3B Induction

**DOI:** 10.1371/journal.pone.0097787

**Published:** 2014-05-23

**Authors:** Kenji Ohba, Koji Ichiyama, Misako Yajima, Nobuhiro Gemma, Masaru Nikaido, Qingqing Wu, PeiPei Chong, Seiichiro Mori, Rain Yamamoto, John Eu Li Wong, Naoki Yamamoto

**Affiliations:** 1 Infectious Disease program, Department of Microbiology, Yong Loo Lin School of Medicine, National University of Singapore, Singapore, Singapore; 2 TOSHIBA Research & Development Center, TOSHIBA Corporation, Kawasaki, Kanagawa, Japan; 3 Materials and Devices Division, TOSHIBA Corporation, Minato-ku, Tokyo, Japan; 4 Department of Biomedical Science, Faculty of Medicine and Health Sciences, Universiti Putra Malaysia, Selangor Darul Ehsan, Malaysia; 5 Pathogen Genomics Center, National Institute of Infectious Diseases, Shinjuku-ku, Tokyo, Japan; 6 Department of Nutrition, Harvard School of Public Health, Boston, Massachusetts, United States of America; 7 Department of Hematology-Medical Oncology, National University Cancer Institute, the Yong Loo Lin School of Medicine, National University of Singapore, Singapore, Singapore; Georgia Regents University, United States of America

## Abstract

High prevalence of infection with high-risk human papilloma virus (HPV) ranging from 25 to 100% (average 31%) was observed in breast cancer (BC) patients in Singapore using novel DNA chip technology. Early stage of BC demonstrated higher HPV positivity, and BC positive for estrogen receptor (ER) showed significantly higher HPV infection rate. This unique association of HPV with BC in vivo prompted us to investigate a possible involvement of HPV in early stages of breast carcinogenesis. Using normal breast epithelial cells stably transfected with HPV-18, we showed apparent upregulation of mRNA for the cytidine deaminase, APOBEC3B (A3B) which is reported to be a source of mutations in BC. HPV-induced A3B overexpression caused significant γH2AX focus formation, and DNA breaks which were cancelled by shRNA to HPV18 E6, E7 and A3B. These results strongly suggest an active involvement of HPV in the early stage of BC carcinogenesis via A3B induction.

## Introduction

Breast cancer (BC) is one of the major health issues faced by women globally, ranking first in mortality (GLOBOCAN 2012: Estimated Cancer Incidence, Mortality and Prevalence Worldwide in 2012). Epidemiology gave the first hints for an involvement of viral agents in specific human cancers [1]. Since the mid-20^th^ century when mouse mammary tumor virus (MMTV) was discovered to cause mammary cancers in mice, some viruses, especially lately HPV, have been suspected to play an etiological role in BC. A number of molecular epidemiological studies conducted have accumulated data relating HPV to BC and indicated that HPV DNA is present at a high frequency in BC samples but is rare in normal breast tissues [2]–[8]. However, results from these studies have been rather varied and sometimes contradictory. Although some authors from these previous studies suggested an etiological role for HPV in BC, no clear explanation had been proffered for the causative mechanism other than the high rate of HPV positivity as the sole reason with a precedent premise that high-risk HPV has been established as a cause of CC. On the other hand, other researchers held an opposing view of these epidemiological results. For instance, Khan et al. concluded that it is unlikely for integrated HPV to be etiologically involved in the development of BC since the viral load was very low (a geometric mean of 5.4 copies per 10^4^ cells) [2]. Many recent reports also showed that HPV genome could be detected in non-genital cancers such as head and neck (oesophageal, laryngeal and tonsil), lung, urothelial, breast and colorectal cancers [9], [10].

HPV is a DNA virus with a circular double stranded configuration, with more than 100 subtypes found to date [11], [12]. Most of the known HPV types cause no symptoms in general, although some types can cause warts, while others are believed to lead to cervical cancer [13] and other forms of genital cancers (vulvar, vaginal, anal, and penile) [14]. The HPV genome is composed of 8 genes (E1, E2, E4, E5, E6, E7, L1 and L2) and a long control region (LCR) which has promoter and replication origin for replication [15]. In the pre-cancerous lesions of cervical cancer, most HPV genomes persist in an episomal state whereas in many high-grade lesions, these viral genomes are found integrated into the host chromosome. Amongst various types of HPVs, 15 types including HPV-16 and HPV-18 are categorized as high-risk oncogenic HPVs based on the fact that they account for over 90% of cervical cancer [16]. E7 protein of high-risk HPVs has a high affinity to human retinoblastoma (Rb) proteins, and this leads to enhanced pRb degradation, resulting in the activation of E2F transcription factors that promote expression of S phase genes [17], [18]. The binding of Rb with E7 leads to inhibition of cell apoptosis through tumor suppressor p53-dependent pathway. Therefore, to overcome apoptosis by p53, HPV exploits another important oncogene product, E6 protein, which can bind p53 for degradation, leading to prevention of cell growth inhibition. E6 and E7 proteins have complementary function and this synergistic effect can efficiently lead to cell cycle progression, anti-apoptotic effect and genomic instability that cause accumulation of cellular mutations over time, and consequent tumor initiation, transformation and progression to cancer [19].

Hosts have means such as innate and adaptive immunities to combat against invasion by various pathogens including bacteria and viruses. Innate immunity is a first guardian, which induces interferon resulting in activation of IFN-stimulated genes (ISGs) [20]. In fact, the APOBEC3 family of cytidine deaminases, which are IFN-inducible, play a key role in the innate immune system against various exogenous viruses as well as endogenous retroviral elements [20]. APOBEC3 enzymes can edit DNA and/or RNA sequences [21]–[23]. However, further studies have revealed that inappropriate expression of APOBEC subgroup seems to act as a genomic mutator that eventually causes cancer [24]–[28]. Indeed, overexpression of other cytidine deaminases, APOBEC1 and activation-induced deaminase (AID) can cause hepatocellular carcinoma and T cell lymphomas in transgenic mice, respectively. Also, several other studies have disclosed expression of AID gene transcripts or proteins in gastric cancer [29] and human lymphoid malignancies [30], [31]. Very recently, A3B has been implicated by several groups independently as a source of mutations in various cancers including breast, bladder, lung, head and neck, and cervical cancers [25]–[28]. It is an indisputable fact that HPV infection is a major risk factor for the development of cervical and head and neck cancers in which the A3B mutation signature is found to be enriched. Here, our aim was to probe whether HPV could be the initiator of breast carcinogenesis through A3B overexpression which leads to excessive mutations, dysregulated cell cycle and subsequent transformation, and thus provide the missing link in the study of the role of HPV in BC causation. We hypothesize that HPV could trigger aberrant expression of A3B in breast epithelial cells which results in genomic instability. We first studied the prevalence of HPV in breast cancer tissue samples from patients, and subsequently tested our hypothesis of HPV’s role in breast carcinogenesis in an *in vitro* system.

## Methods

### DNA Samples

More than 210 fresh frozen breast tumor specimens were obtained from the National University Health System (NUHS) Tissue Repository (TR) with approval from the Institutional Review Board (IRB) at the National University of Singapore (NUS) and NUHS. The specimens were collected from surgical operations in the National University Hospital (NUS) and banked in the NUHS TR.

Genomic DNA (gDNA) was extracted from all above-mentioned BC samples by use of the QIAGEN DNeasy Blood & Tissue Kit (Venlo, Netherlands) and stored at −20°C before use. After extraction, specimens were subjected to DNA microarray array (TOSHIBA, Tokyo, Japan) for the detection of 13 high-risk HPV genomes, according to manufacturer’s instructions.

### HPV Detection Using TOSHIBA Microarray

HPV detection using the TOSHIBA DNA chip was performed according to manufacturer’s instructions [32]. Briefly, 200–500ng of gDNA were denatured at 95°C for 5 min, and then added into each of the 6 loop-mediated isothermal amplification (LAMP) reaction tubes. The LAMP reaction, which amplifies the HPV genome by targeting the L1 region of 13 high-risk HPV subtypes (16, 18, 31, 33, 35, 39, 45, 51, 52, 56, 58, 59 and 68), performed at the following conditions: 65°C for 90 min then 80°C for 5 min. Thereafter, samples from the 6 reaction tubes were collected into one tube, prior to the addition of hybridization buffer and application to the electrochemical DNA chip, which contains specific DNA probes for the L1 region of 13 high-risk HPVs. Hybridization and detection of HPVs were performed by the Genelyzer (TOSHIBA, Tokyo, Japan).

### Constructs

APOBEC3B-promoter (A3B-Pro) was amplified using genomic DNA and specific primers as follows: 5′-ACGGGGTACCGTCTTATCCTTTGTCCATCTCTTTCTGAC-3′, 5′-ACGGGGTACCGTCTTATCCTTTGTCCATCTCTTTCTGAC-3′, and cloned into pGL3 vector (Promega). pUC-HPV16 and pSP-HPV18 plasmids were constructed as described previously [33]. Oligonucleotides for gene specific short-hairpin RNA for APOBEC3B, HPV18-E6 and HPV18-E7 (Table S1) were ordered from Integrated DNA Technologies and cloned into pSuperRetro vector (OligoEngine, WA).

### Cells

293T and MCF10-A cells were obtained from American type tissue culture (ATCC, VA). 293T cells were maintained in 10% FBS supplemented DMEM. MCF10A cells were cultured with 5% horse serum (Lifetech), 20ng/ml rEGF, 100ng/ml Cholera Toxin, 0.5 µg/ml hydrocortisone (SIGMA, MO) and rInsulin (Roche, Upper Bavaria, Germany).

Stably HPV18 infected MCF10-A (MCF10A-HPV18) cells were established by transfection of linear pSP-HPV18 cut by EcoRI followed by limiting dilution at 7 days after transfection to obtain HPV18 infected cells, in which integration of HPV18 genome was confirmed using Alu-PCR.

### Reagents

Anti-HPV18 E7 (8E2) and anti-Flag M2 antibodies were purchased from Abcam (Cambridge, UK) and SIGMA respectively. Anti-γH2AX, γH2AX-Alexa647, β-actin, mouse IgG-HRP and rabbit IgG-HRP antibodies were purchased from Cell Signaling technology.

### Luciferase Assay

Circular or linear HPV plasmid was transfected into 293T and MCF10-A cells with A3B-Pro and TK-Rluc as control. Cells were harvested at 48–72 hrs post-transfection followed by lysis. Luminescence was measured using Dula-Luciserase kit (Promega, WI) and Synergy H1 hybrid Multi-Mode Microplate reader (BioTek, VT).

### RNA Isolation, cDNA Synthesis and qRT–PCR

Patients RNA samples were obtained from the National University Health System (NUHS) Tissue Repository (TR) with approval from the Institutional Review Board (IRB) at the National University of Singapore (NUS) and NUHS. Total RNA from various MCF10-A cells was extracted using RNA easy kit (QIAGEN). Reverse transcription was performed using Goscript reverse transcription system (Promega) after DNase I treatment (Turbo DNA free kit, Lifetech). Quantification of mRNA was performed using SsoAdvanced SYBR Green supermix (Bio-rad, CA) and gene specific primers (Table S1).

### Immunofluorescence

MCF10-A and MCF10A-HPV18 cells were fixed by 4% paraformaldehyde/PBS following washing cells with PBS. Cells were treated with PBS containing 4% normal goat serum (Lifetech), 0.5% Triton-X 100 (SIGMA). E7 or γH2AX proteins were stained by anti-HPV18 E7 (8E2) and anti-mouse IgG-Alexa568 Ab or γH2AX-Alexa647 (CST) followed by DAPI staining. After mounting slides using ProLong Gold Antifade Reagent (Lifetech), cells were observed using Olympus X81 immunofluorescent microscope (Tokyo, Japan).

### Stable A3B, HPVE6 or E7 Knockdown MCF10A Cells

The pSR-shRNA plasmid was transfected with pVSVG plasmid into Plat-E cells and then supernatants were collected at 60 hrs post-transfection after filtration by 0.45 µM syringe-top filter. Cells were infected with retrovirus vectors for 12hrs in presence of polybrene (SIGMA) followed by puromycin (Invivogen, CA) selection (4 µg/ml) for 48hrs. Cells were cultured in presence of 2 µg/ml of puromycin before use. Knockdown of target genes were checked by qRT-PCR as mentioned above.

### Comet Assay

Microscope slides were coated with 1.5% agarose and dried. Low-melting agarose (0.5% in PBS) was combined 1∶1 with MCF10-A and MCF10A-HPV18 cells. Seven-thousand cells were added to coated slides and the cells were lysed overnight in 10mM Tris, 100mM EDTA, 2.5M NaCl, 10%DMSO and 1% Triton X-100 (pH10). Slides were incubated for 20 min in chilled running buffer (300mM NaOH, 1mM EDTA, pH13) then run at 25V for 25 min at 4°C. Gels were neutralized with 400mM Tris-HCl, pH7.5 followed by propidium iodide staining for 1hr. The microgels were allowed to dry and comets were observed by microscope. The % DNA in tail was calculated by Tail DNA intensity/(Head DNA intensity+Tail DNA intensity) × 100.

### Statistical Analysis

Data were analyzed using STATA (version 11.0, Stata Corporation, College Station, TX, USA). Associations between HPV status and various breast cancer parameters and risk factors were tested using either Fisher’s exact test or the Chi-squared test for trend. The tests were all two-sided, and statistical significance was defined as p≤0.05.

## Results

### BC Tissues with Estrogen Receptor (ER) Showed Significantly Higher HPV Prevalence than ER-negative Tumors

**Figure 1:**
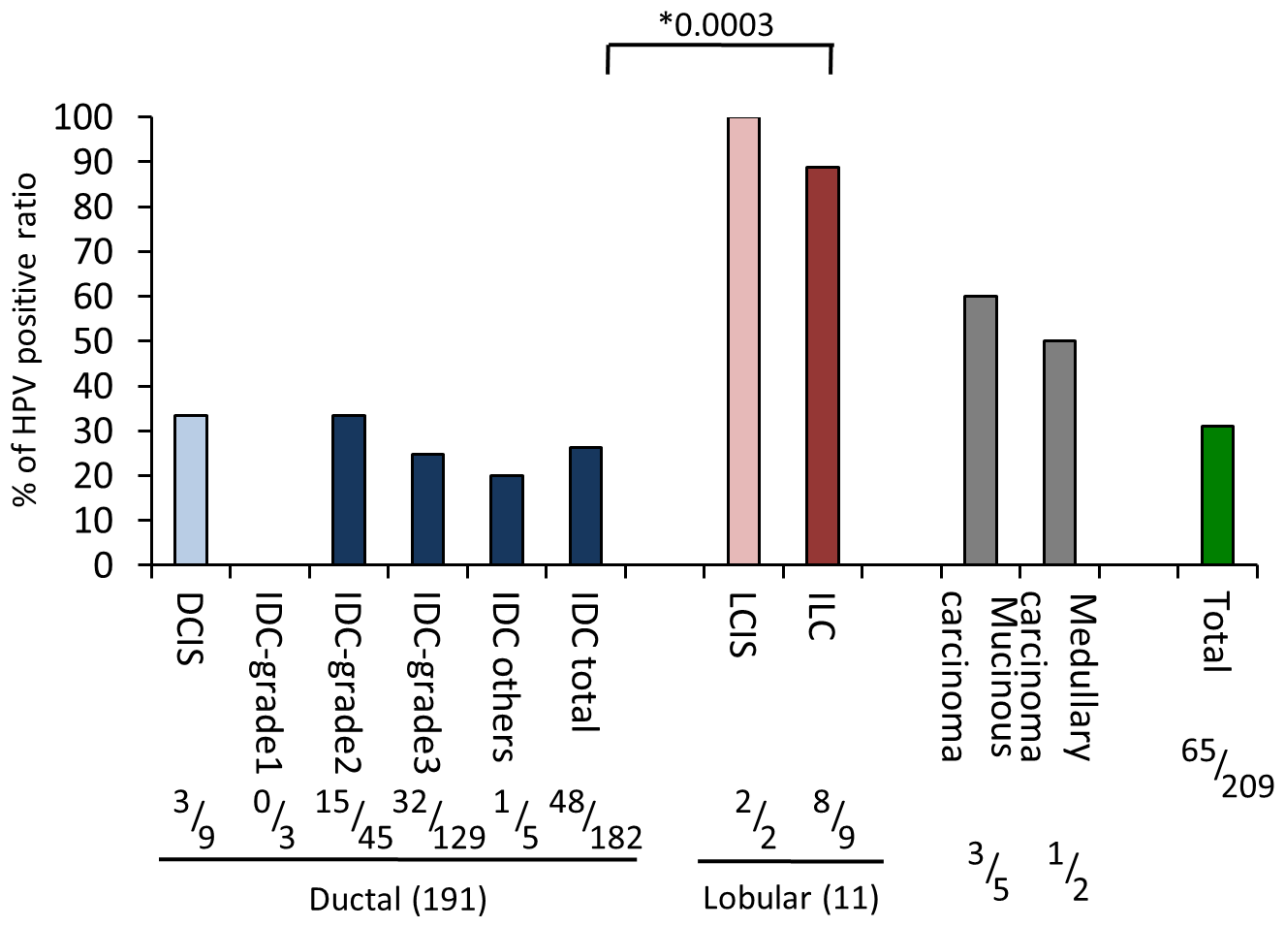
Relationship between HPV status and patholological features. Numbers at the bottom of each bar represent (number of HPV-positives)/(total sample numbers). Difference of HPV prevalence between invasive ductal carcinoma (IDC) and invasive LC (ILC) was statistically significant (*p* = 0.0003). IS; in situ.

We first examined the relationship between HPV status, and pathological and demographic features in 209 female BC cases in Singapore using the DNA chip system and genomic DNA extracted from frozen samples (Figure. 1, Table 1). High-risk HPV DNA was detected in 31% of all BC samples. Association of BC with HPV seemed to depend on age, histological types, and stages of malignancy with the prevalence ranging from about 20% to nearly 100% (Fig. 1 and Table 1). For instance, the prevalence of HPV varied greatly depending on pathological features whereby almost all lobular carcinoma (LC) type of BC cases were positive for HPV even though the numbers were small for both LC in situ (IS) (LCIS) and invasive LC (ILC) (*p* = 0.0003) while that of IDC was lower than 30%. The HPV positivity was lower when the patients were younger, but it seemed to increase with age (*p* for trend = 0.1155). Irrespective of ethnicity, all 3 major groups (Chinese, Malay and Indian) showed essentially a similar HPV prevalence. The patients with a family history of BC and BC at both sides of the breast seemed to show higher HPV positivity suggesting the infectious origin of some types of BC, especially due to high-risk HPVs (Table 1). Importantly, BC cases which are ER-positive showed significantly higher HPV prevalence than ER-negative tumors (*p* = 0.0378) (Table 1).

**Table 1:** Prevalence of the HPV infection according to various demographic and pathological factors in BCs.

	HPV-positive	HPV-negative	Total	*p*-value
	N(%)	N(%)	N(%)	
Age				
<40	1(6.7)	14(93.3)	15	
40–49	12(23.5)	39(76.5)	51	
50–59	17(25.8)	49(74.2)	66	*0.1155
60–69	14(31.8)	30 (68.2)	44	
>70	8(30.8)	18(69.2)	26	
Total	52(25.7)	150(74.3)	202 (100)	
Ethnicity				
Chinese	32(30.8)	72(69.2)	104	
Malay	11(22.4)	38(77.6)	49	
Indian	6(26.1)	17(73.9)	23	**0.445
Others	4(16.7)	20(83.3)	24	
Total	53(26.5)	147(73.5)	200(100)	
Family history				
Yes	7(29.2)	17(70.8)	24	
No	35(25.9)	100(74.1)	135	***0.8026
Unknown	1(6.7)	14(93.3)	15	
Total	43(24.7)	131(75.3)	174(100)	
BC at both sides				
Yes	4(36.4)	7(63.6)	11	
No	42(25.9)	120(74.1)	162	***0.4857
Unknown	0(0.0)	1(100)	1	
Total	46(26.4)	128(73.6)	174(100)	
Estrogen Receptor				
Positive	35(29.8)	83(70.3)	118	
Negative	8(14.5)	47(85.5)	55	$0.0378
Unknown	0(0.0)	1(100)	1	
Total	43(24.7)	131(75.3)	174(100)	
Progesterone Receptor				
Positive	30(26.8)	82(73.3)	112	
Negative	13(21.3)	61(78.7)	61	$0.466
Unknown	0(0.0)	1(100)	1	
Total	43(24.7)	131(75.3)	174(100)	
HER2				
Positive	7(19.4)	29(80.6)	36	
Negative	23(28.8)	57(71.2)	80	$0.3626
Unknown or N/A	13(22.4)	45(77.6)	58	
Total	43(24.7)	131(75.3)	174(100)	
Vascular invasion				
Present	19(28.8)	47(71.2)	66	
Absent	24(24.0)	76(76.0)	100	^$$^0.5875
ND	0(0.0)	8(100)	8	
Total	43(24.7)	131(75.3)	174(100)	
Lymph vessel invasion				
Present	19(29.2)	46(66.7)	65	
Absent	24(24.0)	76(76.0)	100	^$$^0.4727
ND	0(0.0)	9(100)	9	
Total	43(26.4)	131(69.7)	174(100)	
Lymph node metastasis				
Low (1–29%)	15(28.3)	38(71.7)	53	10.2006
Mid (30–59%)	5(26.3)	14(73.7)	19	
High (>60%)	7(28.0)	18(72.0)	25	^2^0.5199
Total (1–100%)	27(27.8)	70(72.2)	97	
No metastasis	13(18.3)	58(81.7)	71	30.3906
Unknown or N/A	1(33.3)	2(66.7)	3	
Total	41(24.0)	130(76.0)	171(100)	^4^0.1993

*Trend test, **Fisher: Chinese v.s. Indian, ***Fisher: Yes v.s. No,

$ Fisher: Positive v.s. Negative, ^$$^Fisher: Present v.s. Absent,

1 Fisher: Meta Low v.s. No meta, ^2^Fisher: meta Mid v.s. No meta,

3 Fisher: Meta high v.s. No meta, ^4^Fisher: Meta total v.s. No meta.

Majority of HPV types found in BC were HPV-16 (47%) and HPV-18 (36%) followed by HPV-35, -56, -59 and -31 subtypes (See Figure S1). We also checked the copy number of HPV in our BC samples using HPV-16 or -18 L1 gene specific quantitative-PCR. As shown in Table S2, most of the cervical cancer samples used as controls had more than 1 copy/cell of HPV-16 or HPV-18 genome, whereas all BC samples at various stages of cancer harbored viral copy numbers at much lower level than 1 copy/cell as reported in previous studies.

### HPV-positive BC Showed Better Prognosis than Virus-negative Tumors

**Figure 2:**
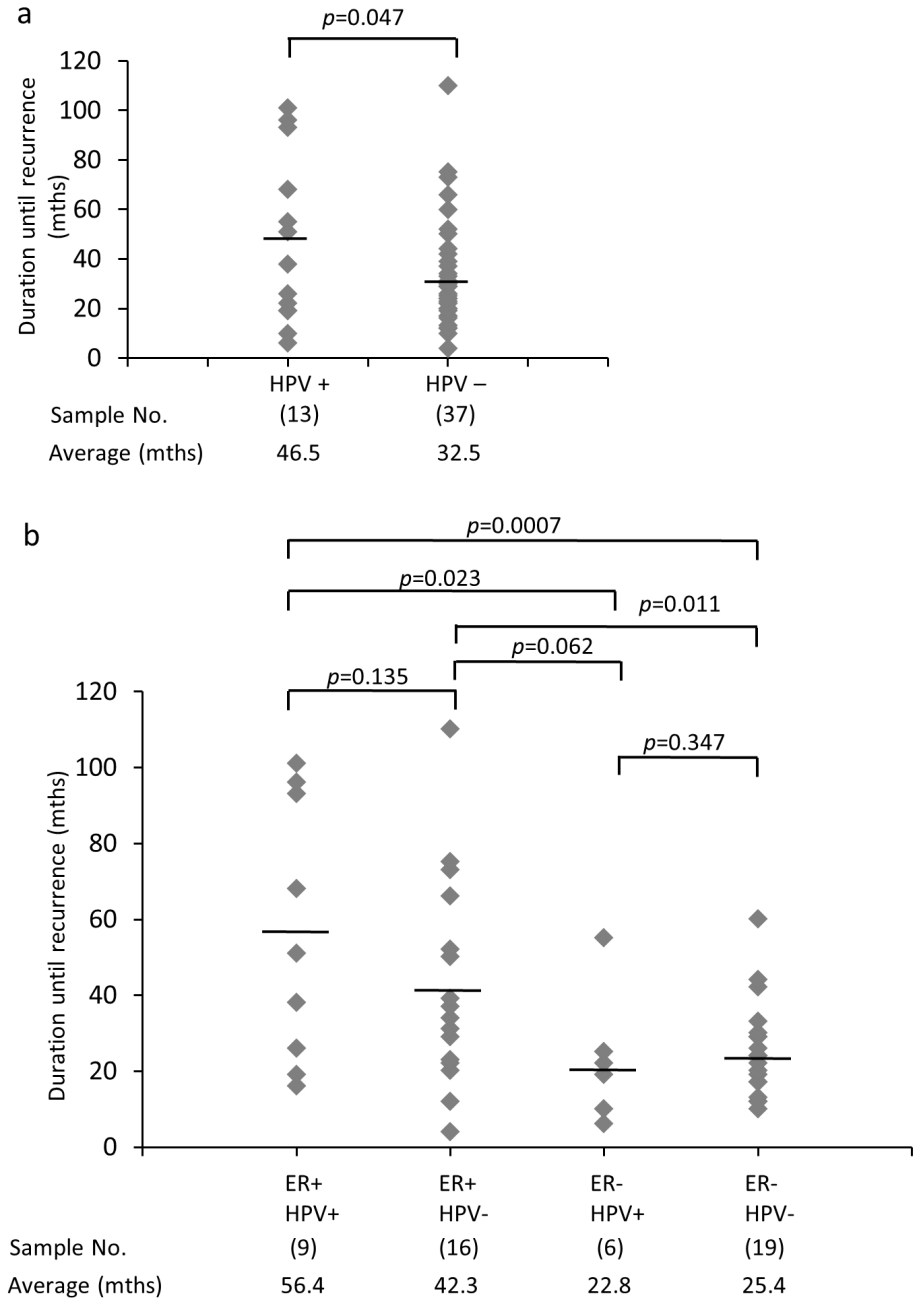
Significant difference of duration until recurrence between HPV-positive and -negative tumors. Fifty BC samples of the patients from whom information were available were compared between HPV-positive and -negative groups (a), and ER/HPV-double positive, ER-single positive, HPV single positive and ER/HPV-double negative groups (b). Numbers in brackets indicate total number of samples for each case, respectively. Student’s t-test was performed, with *p*-values indicated on the graph.

HPV-positive tumors showed significantly longer duration period for recurrence after BC resection as compared to virus-negative tumors (*p* = 0.047) (Fig. 2a). When the same analysis was performed among ER-positive cases exclusively, again HPV-positive tumors showed a longer duration until recurrence as compared to HPV–negative tumors although this was not statistically significant (p = 0.135) (Fig. 2b). Irrespective of HPV status, ER-positive cases apparently showed a longer duration than ER-negative cases. Pre-malignant BCs such as atypical ductal hyperplasia, ductal carcinoma in situ (DCIS) and LCIS highly express ER, and none, low or modest ER-expressing cells are detected in invasive breast carcinoma (IBC) [34], suggesting a possibility that ER-positive BC seems to be at early stage of cancer.

### HPV Induces A3B Expression in Normal Breast Cells

**Figure 3:**
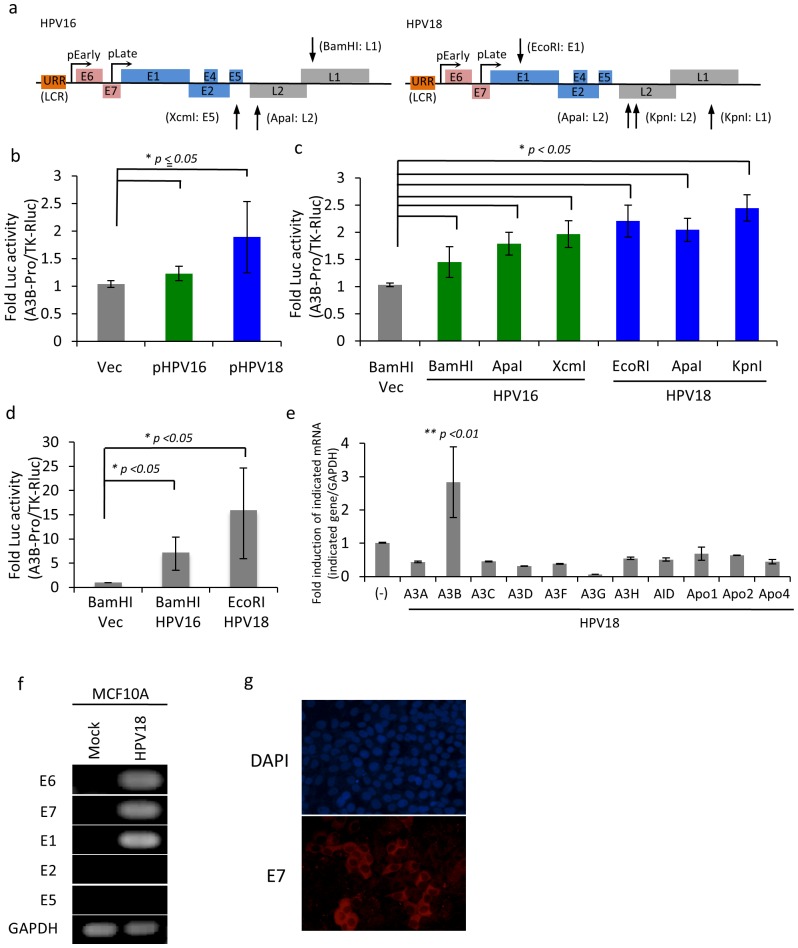
Induction of APOBEC3B by HPV in the normal breast, MCF10-A cells. (a) HPV16 and HPV18 genome structure, and restriction enzyme site position on HPV genome. (b and c) APOBEC3B (A3B) promoter activity. The circular indicated plasmids (b) or same copy number of indicated plasmids digested by restriction enzymes (c) were transfected with A3B-Promoter-luciferase and TK-RLuc into 293T followed by detection of luciferase activity at 48hrs post-transfection. Fold A3B-luciferase activity was normalized by TK-Rluc value. (d) A3B promoter activity at 72hrs in MCF10-A cells as same experiment with panel b and c. (e) Indicated cystidine deaminases mRNA level in MCF10-A and stably HPV18 infected cells. Cells were lysed to extract total RNA, and these RNAs were subjected to quantitative RT-PCR (qRT-PCR). The fold induction of was calculated after normalization by GAPDH mRNA level. (f) RT-PCR for E6, E7, E1 E2 and E5 gene. Cells were lysed to extract total RNA, and its RNA was subjected to conventional RT-PCR. (g) E7 expression in MCF10A-HPV18 cells. Cells were fixed by paraformaldehyde followed by staining of E7 protein and nuclear using anti-HPV18 E7 Ab and DAPI respectively. Values represent the mean ± SD of at least three independent experiments. Student’s t-test was performed, with *p*-values indicated on the graph.

To address whether viral infections could trigger A3B induction and cause mutagenesis of host genomes, we transfected A3B reporter construct together with HPV in either the complete circular form (as a representation of episomal HPV genome) or fragments (as a representation of integrated HPV genome; Fig. 3a) of HPV genomes into 293T cells. Results showed that transfection of circular HPV genome could activate A3B promoter (Fig. 3b). Similarly, cells transfected with fragmented HPV genome showed higher promoter activity than circular HPV genome (Fig. 3c), indicating that E6 and E7 are most likely responsible genes to activate A3B promoter. We also performed the similar experiments with normal breast derived cells, MCF10-A upon transfection with reporter plasmid and fragments of HPV-16 or HPV-18 genome. As shown in Fig. 3d, A3B promoter was strongly activated in MCF10-A cells transfected with HPV genome. Next, we established the MCF10-A cells persistently infected with HPV-18 to see whether A3B gene is consistently activated by HPV infection. HPV-positive cells expressed mRNA for A3B at about 2.5 times more than parental cells calculated based on normalized A3B/GAPDH ratio (Fig. 3e). Interestingly, expression of other cytidine deaminase family genes was in general suppressed (Fig. 3e), suggesting that activation of A3B by HPV seems to be specific.

The results that fragments of HPV18 digested with EcoRI could still activate A3B promoter (Fig. 3a, c and d), and only E6 and E7 intact genes are expressed in persistently HPV18 infected MCF10-A cells (Fig. 3f and g) (although N-terminal of E1 gene is also expressed), suggest that E6 and E7 genes are essential factors to induce A3B expression. Next, 293T cells were transfected with E6 or E7 expression plasmid together with reporter genes to address whether these viral oncogenes were responsible for A3B induction. The results showed that though weakly but still significantly, both E6 and E7 were able to induce A3B expression (Fig. S2), indicating that E6 and E7 are responsible in upregulating A3B expression. These results show that HPV infection in breast cells can specifically activate A3B and its induction seems to be cooperatively regulated by E6 and E7.

### HPV-dependent A3B Activation Triggers Genomic Instability in MCF 10-A Cells

**Figure 4:**
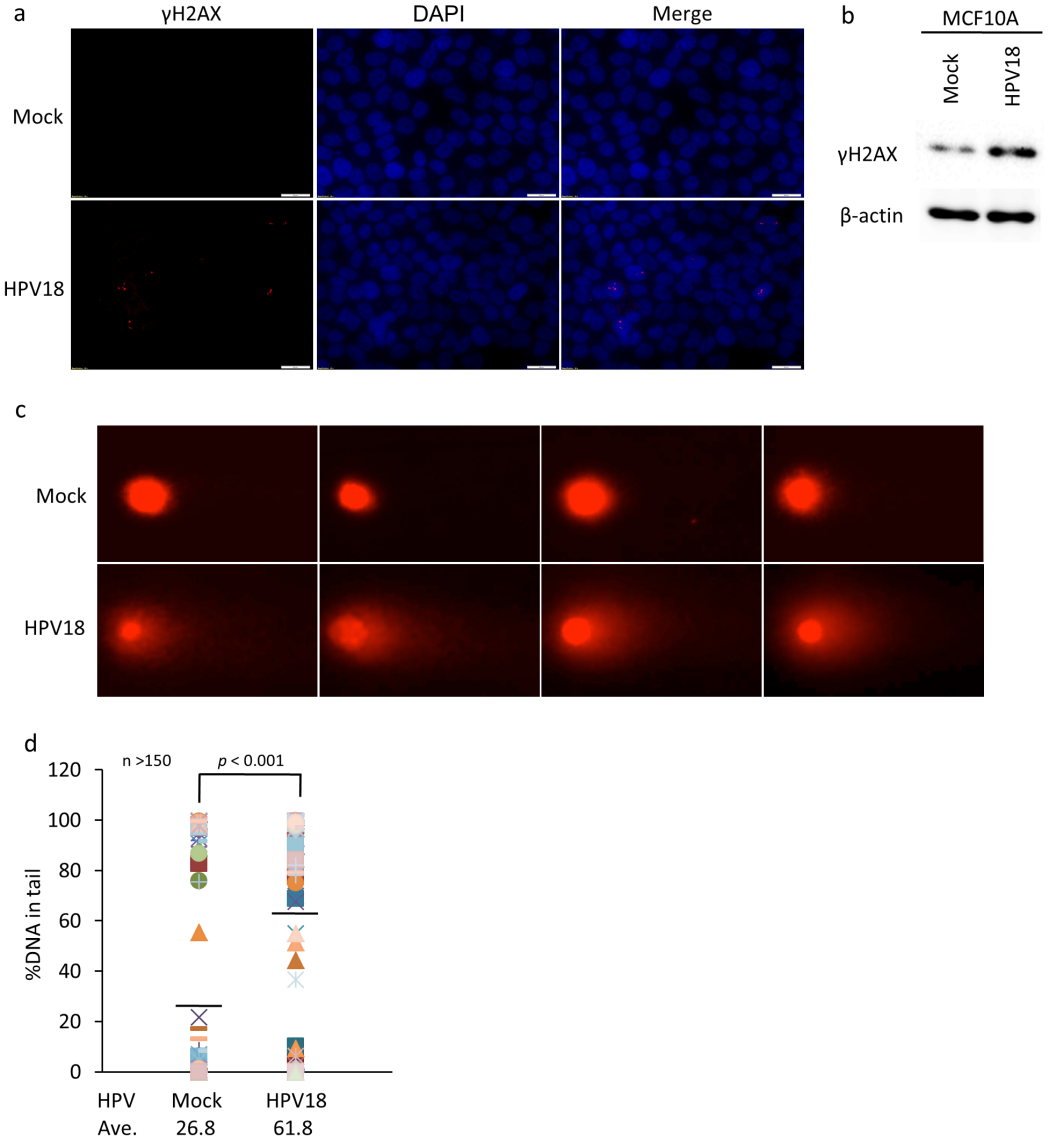
A3B activation by HPV infection increases genome instability. (a) γH2AX immunofluorescence. Cells were fixed by 4% paraformaldehyde followed by staining of γH2AX protein and nuclear using anti- γH2AX Ab and DAPI respectively. (b) γH2AX western blot. Cells were lysed and subjected to western blot. The γH2AX and β-actin were detected using anti- γH2AX and β-actin Ab. (c) Comet assay. Cells were seeded onto agarose-coated slide glass after mixing with 1.5% agarose followed by lysis. Slides were subjected to electrophoresis and then genomic DNA was stained by PI. (d) Statistical analysis for panel C. Student’s t-test was performed, with *p*-values indicated on the graph.

Phosphorylated-Histone 2AX (known as γH2AX), which is widely known as a marker of DNA damage for cancerous phenotype, was significantly increased in MCF10-A persistently infected with HPV18 compared to mock infected cells as revealed by immuno-staining (Fig. 4a) and Western blotting (Fig. 4b). With a corresponding increase of γH2AX, single/double-strand breaks were apparently and strongly induced in persistently HPV18 infected MCF10A cells by the comet assay (Fig. 4c and d). These results indicate that A3B activation through HPV infection seems to induce genomic instability and DNA breaks to initiate tumor.

### Knockdown of A3B, E6 and E7 Abrogated HPV-induced Genomic Instability

**Figure 5:**
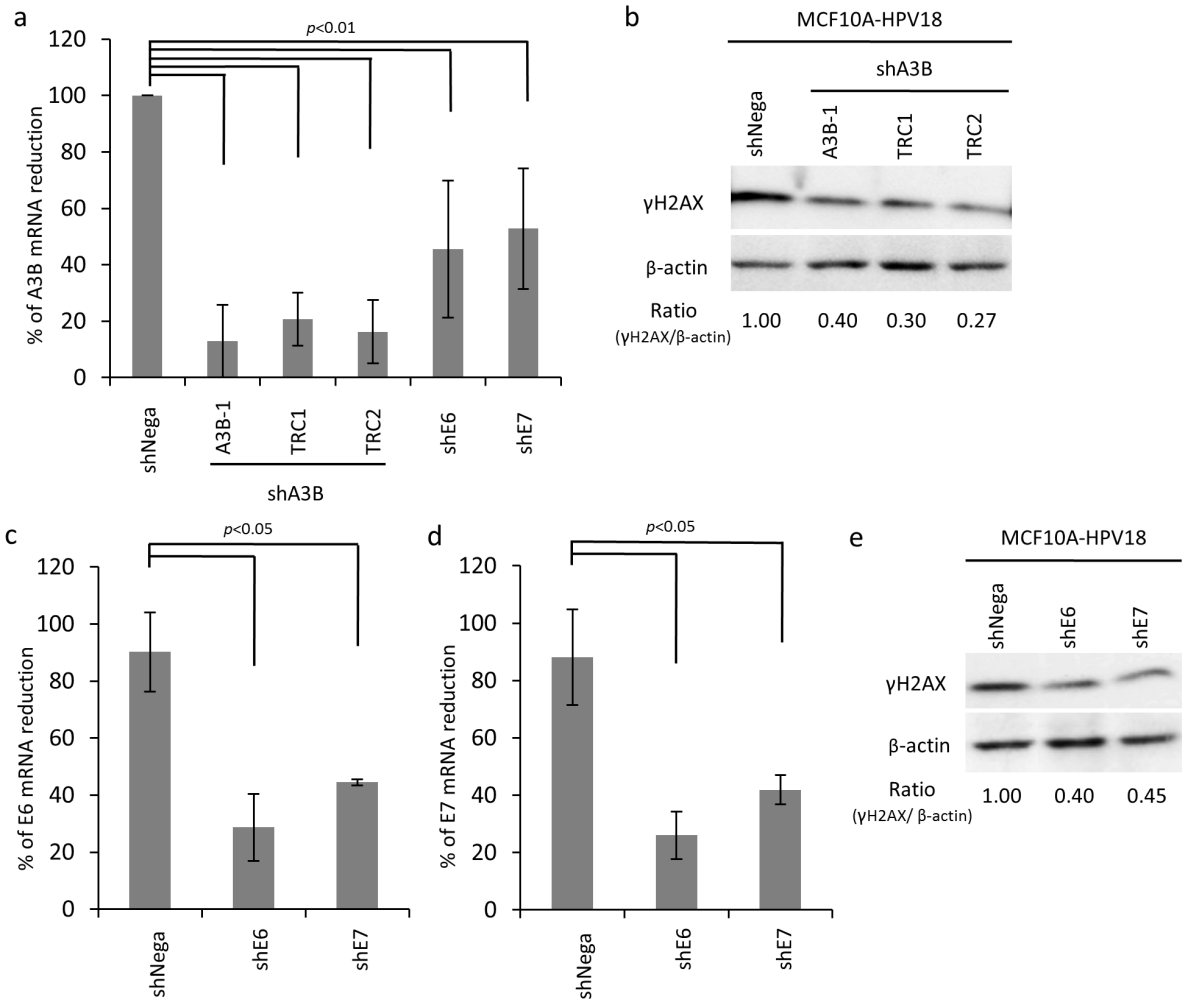
Abrogation of HPV-induced cancer phenotypes by shRNA against HPV E6, E7 and A3B. (a) A3B mRNA level in stable A3B, E6 or E7-knockdown MCF10A-HPV18 cells. A3B, E6 or E7 stably knocked down cells were established using shRNA retroviral vector. Total RNA was extracted from cells and then subjected to qRT-PCR. (b) γH2AX level in stable A3B-knockdown MCF10A-HPV18 cells. Cells were lysed after selection using puromycin and then subjected to western blot. The γH2AX and β-actin were detected using anti-γH2AX and β-actin Ab. The number at bottom of panel shows band intensity ratio after normalized by β-actin level. (c and d) HPV-18 E6 or E7 mRNA level in stable E6 or E7-knockdown MCF10A-HPV18 cells. Total RNA was extracted and then subjected to qRT-PCR. (e) γH2AX level in stable HPV18 E6 or E7-knockdown MCF10A- HPV18 cells. Cells were lysed after selection using puromycin, and then subjected to western blot. The γH2AX and β-actin were detected using anti-γH2AX and β-actin Ab. The number at bottom of panel shows band intensity ratio after normalization by β-actin level. Values represent the mean ± SD of at least three independent experiments. Student’s t-test was performed, with *p*-values indicated on the graph.

We next conducted knockdown experiments for A3B, E6 and E7 using gene specific shRNA to address our hypothesis. Various A3B-specific shRNAs could decrease A3B expression (Fig. 5a), and the silencing of A3B alone abrogated γH2AX induction (Fig. 5b). Both shRNA for E6 and E7 could simultaneously decrease E6 and E7 expression due to transcription of E6 and E7 genes from the same mRNA (Fig. 5c and d), and reduction of E6 and E7 expression resulted in suppression of γH2AX induction (Fig. 5e). More importantly, silencing of E6 and E7 also cancelled A3B induction (Fig. 5a), indicating that A3B is apparently activated through HPV18 E6 and E7, and its activation renders cells cancerous as evidenced by genomic instability condition. However, knockdown of E6 and E7 still showed significant further reduction of γH2AX even in stable A3B-knockdown MCF10A-HPV18 cells, in which more than 85–90% of A3B expression were suppressed (Fig. S3). These results suggest that DNA damage and γH2AX could be induced through two possible pathways; 1) HPV18 E6/E7-A3B (A3B-dependent) and 2) HPVE6/E7 direct (A3B-independent) pathway (Fig. S4).

### HPV Infection and A3B Upregulation Seem to be Correlated in BC Patients’ Samples

**Figure 6:**
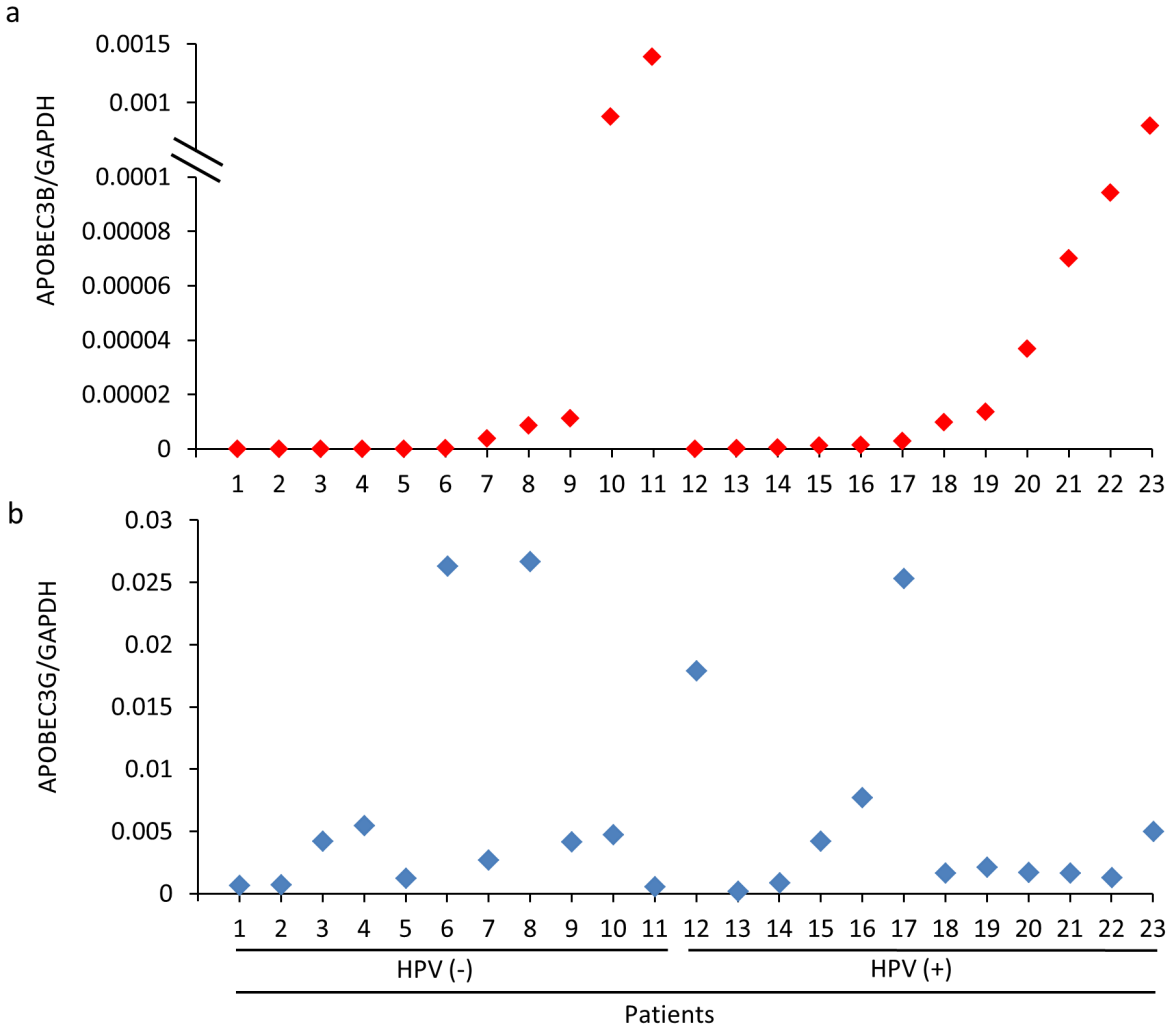
Correlation of A3B expression level and HPV infection in BC patients. Total RNA from BC patients were obtained from NUHS TR. Samples were subjected to quantitative RT-PCR (qRT-PCR). The data of A3B (a) and A3G (b) were shown after normalization by GAPDH mRNA level. The number at the bottom of graph indicates the number of patients. Patient 1–11 are HPV-negative, patient 12–23 are HPV-positive.

Finally, we checked whether A3B upregulation is associated with HPV infection using BC samples from patients. According to results from Fig. 1 and table 1, HPV-negative (n = 11) and -positive (n = 12) BC were randomly selected, and then A3B and A3G mRNA level were monitored. As shown in Fig. 6 a and b, result showed tendency that A3B expression level seems to be correlated with HPV infection although statistical significance could not be obtained. In contrast, there was no correlation at all between A3G and HPV status consistent with a previous report showing that there is no association between A3G expression and BC [25]. Taken together, our results implicate that A3B, which is reported to be a specific source of mutation in BC, is actually induced by HPV infection, suggesting that HPV is likely to be one of the initiators for at least a certain portion of BCs.

## Discussion

To address whether HPV is directly involved in carcinogenesis of BC, we first conducted a molecular epidemiological analysis using patients’ samples. Our results showed the evidence of high-risk HPVs infection in BC patients unambiguously among the different ethnic populations (Table 1). A possible preponderance of lobular histological types (LC) with HPV infection was noted. Most prevalent subtypes were HPV-16 and -18, and other types of HPV such as HPV-35, -56, -59 and -31 followed by other minor subtypes.

Our data showed that tumor cells with ER significantly correlated with high HPV infection (Table 1). The observation raises very intriguing possibilities that 1) estrogen can directly affect transcription of HPV E6/E7 oncogenes in BC system, and 2) ER-positive normal cells could be an initial target of HPVs because these cells are activated by estrogen growth signaling (Fig. S4). A study in murine model of cervical cancer suggested that HPV infection alone is not sufficient to lead to cervical carcinogenesis, and that estrogen is required [35]. Moreover, the synergy between estrogen and p53 insufficiency by E6 protein is important for carcinogenesis of cervical and breast cancer in mice model [36]. In addition, HPV-positive and ER/HPV-positive BCs show longer duration until recurrence than others (Fig. 2). Etiological study reports that pre-malignant BCs highly express ER [34], indicating that ER-positive BCs represent early stage of cancer compared to ER-negative BCs. Therefore, it is quite reasonable that ER-positive BCs tend to show better prognosis even if the advantage that chemotherapy is available for ER-positive BCs is not considered. Collectively, these data indicate that HPV may have some role in the early stage of BCs, and suggest the relationship between estrogen and HPV E6/E7-dependent BC initiation.

We confirmed several past reports showing that HPV, especially high-risk HPV, was detected from the samples of BC in vivo. Our data is also consistent with those from previous studies by Khan et al. which found that HPV load is very low in BC samples, being far lower than 1 copy/cell [2] (Table S2). However, it is well known that some infectious agents (e.g. Helicobacter pylori for gastric cancer and hepatitis viruses for hepatocellular carcinoma) act as indirect carcinogens, without persistence of their genes within the respective cancer cells [1]. Even in HPV, some cutaneous types are attributed as indirect carcinogens [1]. Another important fact to support HPV as possible cause of BC is high-risk HPV E6 and E7 are used as the most efficient and reproducible model for human mammary epithelial carcinogenesis in vitro. These data prompted us to test an intriguing possibility that HPV might act predominantly at earlier but not so significantly at later steps of breast carcinogenesis. HPV E6 and E7 proteins are oncogenic per se and their synergistic effects can efficiently lead to cell cycle progression, anti-apoptotic effect and genomic instability. Indeed, previous reports have demonstrated that HPV can induce γH2AX and DNA breaks [19], [37]. During our investigations seeking for possible DNA mutators in the host inducible by HPV, we encountered striking data suggesting a model in which A3B-catalysed deamination provides a chronic source of DNA damage in BC that could mutate TP53 [25]. Simultaneously, several groups independently reported using a novel analytical approach that A3B is a likely source of mutations in most major human cancers including breast carcinoma, bladder, lung, cervical, and head and neck cancers [26], [38]. Importantly, HPV is attributed as a cause of the latter 2 types of cancers. Thus, we investigated the relationship between A3B mutagenesis and HPV infection in breast cells. Our results clearly show that HPV infection induces overexpression of A3B (Fig. 3) and infected cells represent more malignant phenotype than parental cells (Fig. 4). These malignant phenotypes were largely abrogated when A3B was knocked down in HPV-infected cells (Fig. 5). Hence, it is indicated that HPV effect is exerted mainly through induction of this enzyme and is more indirect in BC oncogenesis.

We found a positive correlation between A3B expression and HPV infection even in primary BC samples (Fig. 6) although the correlation did not reach statistical significance, most probably due to the small sample numbers. In this regard, it is notable that EBV, which shares many similarities with HPV, is also reported to be highly associated with BC in vivo epidemiologically [7], [39]. Our preliminary studies indicate that EBV can induce a wider range of AID/APOBEC family proteins more efficiently than HPV (Ohba, unpublished results). It is thus possible that high expression of A3B seen in HPV-negative samples is caused by other viruses such as EBV. The experiments seeking for possible involvement of EBV in BC and to inquire whether HPV-infected cells that express high levels of A3B show more mutations than uninfected parental cells are underway. All these data support our contention that HPV plays an important role mainly at initial step of breast carcinogenesis whereas the same virus seems to play the role in both initiation and maintenance in CC oncogenesis. Once the cells undergo critical mutation at certain host genes initially, persistent effect of A3B and its inducing agent HPV are no longer required given the mutator role of A3B, or their continued presence in the tissue possibly is selected against. Therefore, proof that DNA deaminases induce human cancer is at best indirect [40].

It has been suggested for a long time that inflammation and cancer share some basic mechanisms [41]. Especially, an important aspect to be considered is based on the fact that A3B is the product of one of the interferon-inducible genes (ISG) [20]–[23]. Accidental induction of deaminases can result in mutations in key target genes such as tumor suppressors and oncogenes. It is reported that estrogen and inflammatory cytokines, such as interferon, interleukin-6, and tumor necrosis factor can induce AID/APOBEC ptoteins through NF-κB [40], [42]–[46]. As such, would there be an absolute requirement for HPV specifically as compared to other infectious agents in BC carcinogenesis? As compared to most other conventional viruses, HPV (and some other concogenic viruses) is apparently unique in its ubiquitousness and oncogenicity with viral oncogenes. A more important feature of human oncogenic viruses is their modest replicative ability so that their cytotoxicity and interferon inducing ability are relatively weak. This rather modest ability to induce AID/APOBEC family protein of HPV in cells may well explain long latency and requirement of additional factors in generation of human cancer including BC. If this assumption is correct, it is also important to address a possible involvement of EBV or its synergy with HPV in BC carcinogenesis because of its high epidemiological association with BC *in vivo* [7], [39]. In this context, some BC-related clinical conditions such as inflammatory BC definitely require similar studies on possibility of the involvement of infectious agents including HPV, EBV and other microbes, too.

In present study, we showed that HPV seems to be possible initiator for BCs development through A3B activation leading to genomic instability. However, there are still several pathways for induction of genomic instability and tumor initiation. Among these pathways, oxidative stress (OS) including reactive oxygen species (ROS) is one of the sources to induce DNA damage including DNA double-strand breaks causing cancer initiation [47]–[49]. Some infectious agents such as hepatitis B, C and helicobacter pylori can indeed induce OS during infection [47], [50]. Recently, some reports attempted to show the involvement of OS in HPV-dependent carcinogenesis [51]–[53]. However, the role of OS in HPV infection is still unclear, especially in tumor initiation step. There are various possibilities that A3B and OS act independently, concomitantly, cooperatively or even synergistically to induce tumor initiation during HPV infection. Further analysis is required to elucidate whole tumor initiation mechanism by HPV.

In conclusion, we propose here a provocative carcinogenic mechanism of BC that aberrant A3B expression caused by HPV infection might be a mechanism of mutation accumulation in the breast epithelium during HPV-associated breast carcinogenesis which possibly occurs in the early stage of multi-step, multi-factorial carcinogenesis. Recent genome-wide analysis results on broad-spectrum cancer also show the relevance of our contention [27], [28]. If this model turns out to be at all feasible, reevaluation of several other HPV-associated non-genital cancer systems such as those in lung, colon, bladder, and so on is warranted since these tumors represent a significant reminiscence to BC in terms of high infection rate but low copy numbers of high-risk HPV in cancer tissue [9]. Moreover, the same idea could be applied even to the other types of human cancers induced by other oncogenic DNA viruses such as EBV and human polyomaviruses, the roles of which are poorly appreciated so far. Eventually, the role of HPV in development of BC and other HPV-associated non-genital cancers will be ascertained by monitoring the effect on the disease prevalence in women by cervical cancer vaccinations against high-risk HPV types in comparison to women who had not been vaccinated.

## Supporting Information

Figure S1 **HPV genotypes analysis in BC tissues.** Thirteen high-risk HPVs (16, 18, 31, 33, 35, 39, 45, 51, 52, 56, 58, 59 and 68) in derived from fresh BC tissues were detected in gDNAs using the TOSHIBA DNA chip. Percentage of HPV types were analysed with HPV positive samples (n = 217).
(TIF)

Figure S2 **Dose-dependent APOBEC3B promoter activation by HPVE6 and E7.** (a and b) APOBEC3B (A3B) promoter activity. The indicated dose of E6 (b) and Flag-E7 (c) plasmids were transfected with A3B-Promoter-luciferase and TK-RLuc into 293T followed by detection of luciferase activity at 48hrs post-transfection. Fold A3B-luciferase activity was normalized by TK-Rluc value. Values represent the mean ± SD of three independent experiments. (c) Dose-dependent expression of Flag-E7. Cells were lysed, and then subjected to western blot. The Flag-E7 and β-actin were detected using anti-Flag M2 and β-actin Ab.
(TIF)

Figure S3 **Additional γH2AX reduction by shHPV18 E6 or E7 in stable A3B-knockdown MCF10A-HPV18 cells.** The γH2AX level in A3B and HPV18 E6 or E7-knockdown MCF10A-HPV18 cells. Stable A3B-knockdown MCF10A-HPV18 cells were transfected with HPV18 E6 or E7 shRNA plasmid. Cells were lysed at 72 hrs after transfection, and then subjected to western blot. The γH2AX and β-actin were detected using anti-γH2AX and β-actin Ab. The number at bottom of panel shows band intensity ratio after normalization by β-actin level.
(TIF)

Figure S4 **Possible BC initiation mechanism by HPV.** The overall possible scheme of BC initiation mechanism by HPV. 1) HPV is infected to cells, replicates viral proteins and genome and then produces progeny viruses. 2) Estrogen receptor augments HPV replication. 3) A3B production is induced by E6 and E7 proteins derived from HPV episome or integrated genome that results in persistent HPV infection. 4) Induced A3B by E6/E7 and E6/E7 proteins themselves augment genomic instability such as DNA breaks resulting in γH2AX activation. 5) Genomic instability accumulates mutation in host genome and renders cells cancerous. 6) Simultaneously, E6/E7 interfere p53/Rb function respectively to prevent apoptosis and promote cell cycle progression. 7) Those molecular mechanisms cooperatively generate tumor.
(TIF)

Table S1 **Primers and Oligonucleotides information.** The information of RT-PCR primers and shRNA sequences used in present study.
(DOC)

Table S2 **HPV genome copy number in BC.** HPV16 and 18 viral DNA in breast cancer (BC) and cervical cancer (CC) tissues were quantified by real-time quantitative polymerase chain reaction (PCR) using a TaqMan probes as mentioned in. Viral DNA was calculated based on the standard curve of control DNA.
(DOC)

Text S1 Supporting information text describes materials and methods for all of supporting figures and tables.
(DOC)
