# Supplementary figures and images for: In Vivo and In Vitro Studies Suggest a Possible Involvement of HPV Infection in the Early Stage of Breast Carcinogenesis via APOBEC3B Induction
Source: PLoS One. 2014 May 23;9(5):e97787. doi: 10.1371/journal.pone.0097787 (PMC4032256; doi:10.1371/journal.pone.0097787)

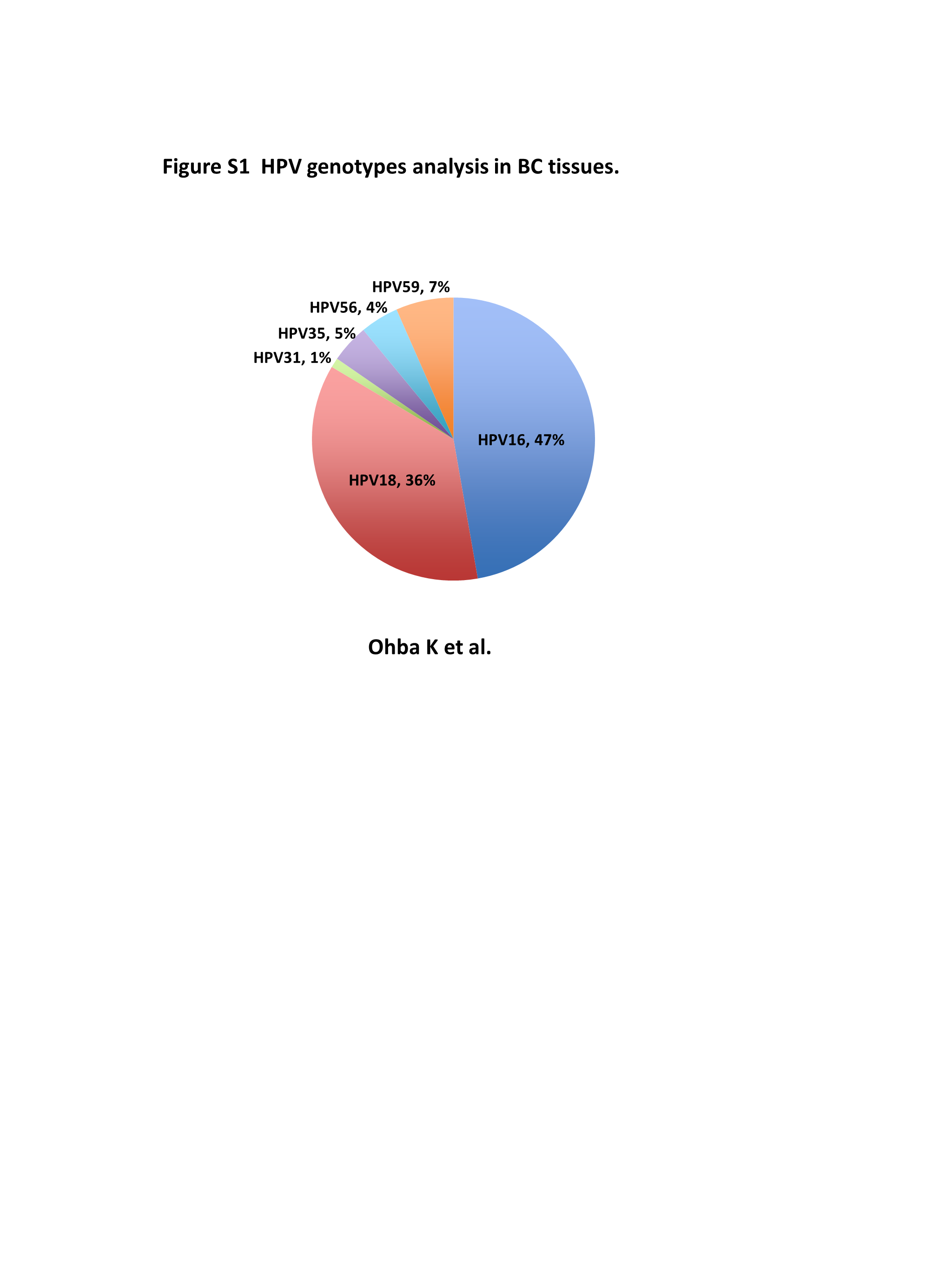

Supplement: Figure S1 — HPV genotypes analysis in BC tissues. Thirteen high-risk HPVs (16, 18, 31, 33, 35, 39, 45, 51, 52, 56, 58, 59 and 68) in derived from fresh BC tissues were detected in gDNAs using the TOSHIBA DNA chip. Percentage of HPV types were analysed with HPV positive samples (n = 217). (TIF) [file pone.0097787.s001.tif]

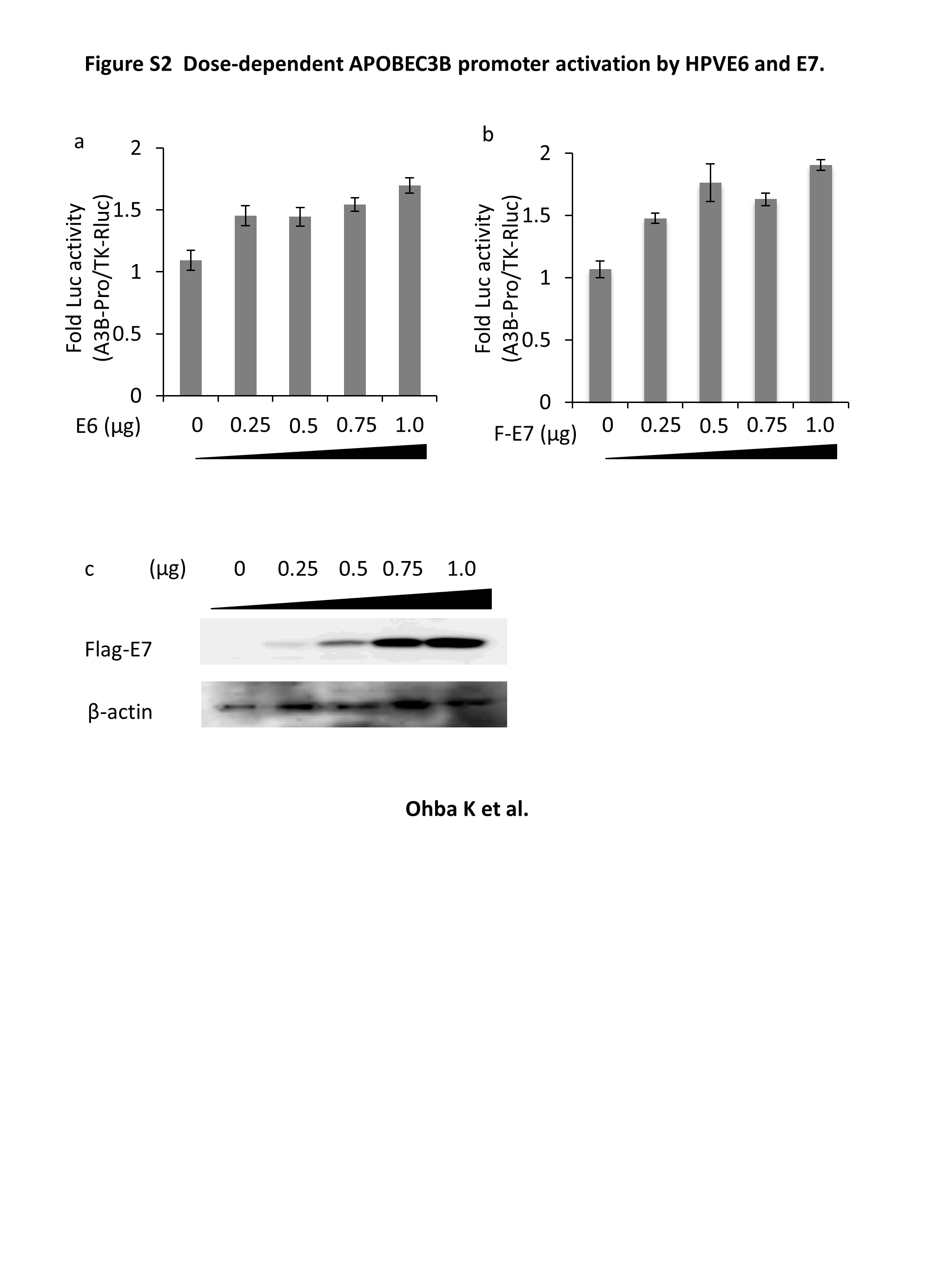

Supplement: Figure S2 — Dose-dependent APOBEC3B promoter activation by HPVE6 and E7. (a and b) APOBEC3B (A3B) promoter activity. The indicated dose of E6 (b) and Flag-E7 (c) plasmids were transfected with A3B-Promoter-luciferase and TK-RLuc into 293T followed by detection of luciferase activity at 48hrs post-transfection. Fold A3B-luciferase activity was normalized by TK-Rluc value. Values represent the mean ± SD of three independent experiments. (c) Dose-dependent expression of Flag-E7. Cells were lysed, and then subjected to western blot. The Flag-E7 and β-actin were detected using anti-Flag M2 and β-actin Ab. (TIF) [file pone.0097787.s002.tif]

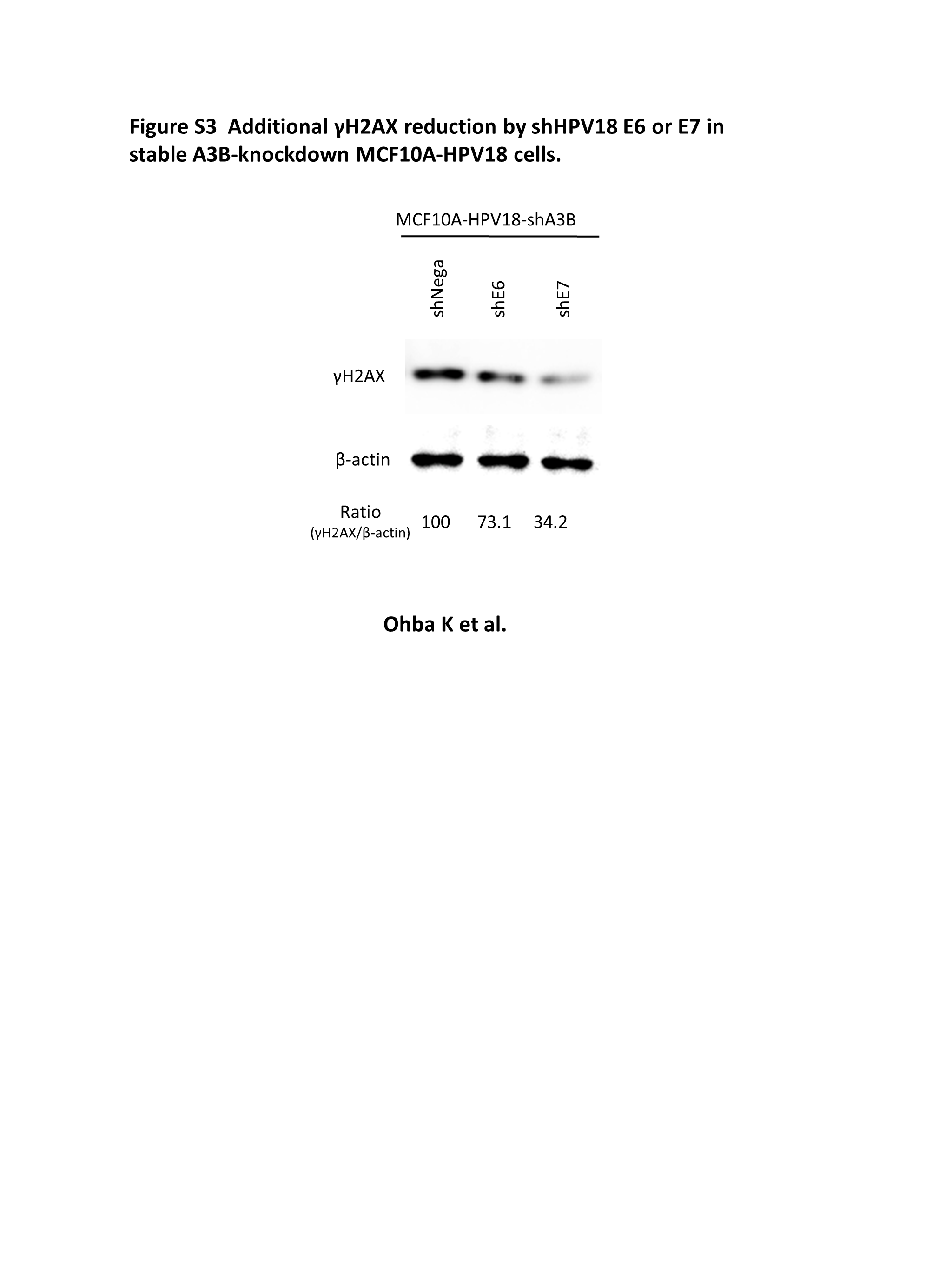

Supplement: Figure S3 — Additional γH2AX reduction by shHPV18 E6 or E7 in stable A3B-knockdown MCF10A-HPV18 cells. The γH2AX level in A3B and HPV18 E6 or E7-knockdown MCF10A-HPV18 cells. Stable A3B-knockdown MCF10A-HPV18 cells were transfected with HPV18 E6 or E7 shRNA plasmid. Cells were lysed at 72 hrs after transfection, and then subjected to western blot. The γH2AX and β-actin were detected using anti-γH2AX and β-actin Ab. The number at bottom of panel shows band intensity ratio after normalization by β-actin level. (TIF) [file pone.0097787.s003.tif]

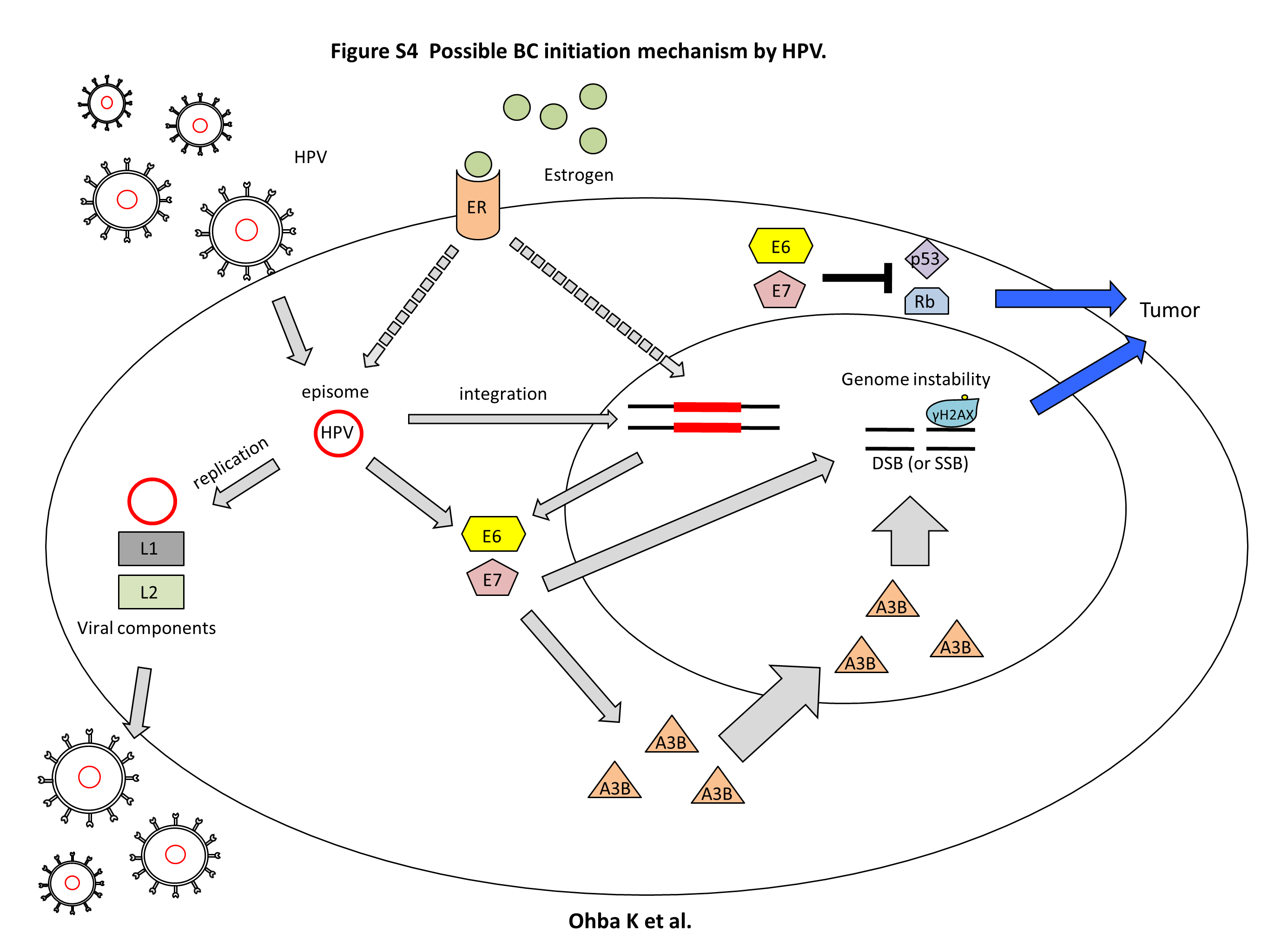

Supplement: Figure S4 — Possible BC initiation mechanism by HPV. The overall possible scheme of BC initiation mechanism by HPV. 1) HPV is infected to cells, replicates viral proteins and genome and then produces progeny viruses. 2) Estrogen receptor augments HPV replication. 3) A3B production is induced by E6 and E7 proteins derived from HPV episome or integrated genome that results in persistent HPV infection. 4) Induced A3B by E6/E7 and E6/E7 proteins themselves augment genomic instability such as DNA breaks resulting in γH2AX activation. 5) Genomic instability accumulates mutation in host genome and renders cells cancerous. 6) Simultaneously, E6/E7 interfere p53/Rb function respectively to prevent apoptosis and promote cell cycle progression. 7) Those molecular mechanisms cooperatively generate tumor. (TIF) [file pone.0097787.s004.tif]
