# Supplementary material for: In Vivo and In Vitro Studies Suggest a Possible Involvement of HPV Infection in the Early Stage of Breast Carcinogenesis via APOBEC3B Induction
Source: PLoS One. 2014 May 23;9(5):e97787. doi: 10.1371/journal.pone.0097787 (PMC4032256; doi:10.1371/journal.pone.0097787)
Supplement: Table S1 — Primers and Oligonucleotides information. The information of RT-PCR primers and shRNA sequences used in present study. (DOC) [file pone.0097787.s005.doc]

**Table S1** Primers and Oligonucleotides information

| RT-PCR primers |  |
| --- | --- |
| APOBEC3A | 5’-GAGAAGGGACAAGCACATGG-3’ |
|  | 5’-TGGATCCATCAAGTGTCTGG-3’ |
| APOBEC3B | 5’-GACCCTTTGGTCCTTCGAC-3’ |
|  | 5’-GCACAGCCCCAGGAGAAG-3’ |
| APOBEC3C | 5’-AGCGCTTCAGAAAAGAGTGG-3’ |
|  | 5’-AAGTTTCGTTCCGATCGTTG-3’ |
| APOBEC3D | 5’-ACCCAAACGTCAGTCGAATC-3’ |
|  | 5’-CACATTTCTGCGTGGTTCTC-3’ |
| APOBEC3F | 5’-CCGTTTGGACGCAAAGAT-3’ |
|  | 5’-CCAGGTGATCTGGAAACACTT-3’ |
| APOBEC3G | 5’-CCGAGGACCCGAAGGTTAC-3’ |
|  | 5’-TCCAACAGTGCTGAAATTCG-3’ |
| APOBEC3H | 5’-AGCTGTGGCCAGAAGCAC-3’ |
|  | 5’-CGGAATGTTTCGGCTGTT-3’ |
| AID | 5’-GACTTTGGTTATCTTCGCAATAAGA-3’ |
|  | 5’-AGGTCCCAGTCCGAGATGTA-3’ |
| APOBEC1 | 5’-GGGACCTTGTTAACAGTGGAGT-3’ |
|  | 5’-CCAGGTGGGTAGTTGACAAAA-3’ |
| APOBEC2 | 5’-AAGTAGGGCAACTGGGCTTT-3’ |
|  | 5’-GGCTGTACATGTCATTGCTGTC-3’ |
| APOBEC4 | 5’-TTCTAACACCTGGAATGTGATCC-3’ |
|  | 5’-TTTACTGTCTTCTAGCTGCAAACC-3’ |
| GAPDH | 5’-GAAGGTGAAGGTCGGAGTC-3’ |
|  | 5’-GAAGATGGTGATGGGATTTC-3’ |
| shRNA sequences |  |
| shA3B-1 | 5’-gatccccGGATGTATCGAGACACATTttcaagagaAATGTGTCTCGATACATCCtttttggaaa-3’ |
|  | 5’-AGCTtttccaaaaaGGATGTATCGAGACACATTtctcttgaaAATGTGTCTCGATACATCCggg-3’ |
| shA3B TRC1 | 5’-gatccccGCTCAAATCTCCTTTGGGACAttcaagagaTGTCCCAAAGGAGATTTGAGCttttta-3’ |
|  | 5’-agcttaaaaaGCTCAAATCTCCTTTGGGACAtctcttgaaTGTCCCAAAGGAGATTTGAGCggg-3’ |
| shA3B TRC2 | 5’-gatccccGCAAAGCAATGTGCTCCTGATttcaagagaATCAGGAGCACATTGCTTTGCttttta-3’ |
|  | 5’-agcttaaaaaGCAAAGCAATGTGCTCCTGATtctcttgaaATCAGGAGCACATTGCTTTGCggg-3’ |
| shHPV18 E6 | 5’-GATCCgTAGGTATTTGAATTTGCATttcaagagaATGCAAATTCAAATACCTAttttttggaaa-3’ |
|  | 5’-AGCTtttccaaaaaaTAGGTATTTGAATTTGCATtctcttgaaATGCAAATTCAAATACCTAcG-3’ |
| shHPV18 E7 | 5’-gatccccGACCTTCTATGTCACGAGCAAttcaagagaTTGCTCGTGACATAGAAGGTCtttttggaaa-3’ |
|  | 5’-AGCTtttccaaaaaGACCTTCTATGTCACGAGCAAtctcttgaaTTGCTCGTGACATAGAAGGTCggg-3’ |
| shNegative | 5’-gatccccGCTCCCGTGAATTGGAATCCTttcaagagaAGGATTCCAATTCACGGGAGCttttta-3’ |
|  | 5’-AGCTtaaaaaGCTCCCGTGAATTGGAATCCTtctcttgaaAGGATTCCAATTCACGGGAGCggg-3’ |
