# Supplementary material for: In Vivo and In Vitro Studies Suggest a Possible Involvement of HPV Infection in the Early Stage of Breast Carcinogenesis via APOBEC3B Induction
Source: PLoS One. 2014 May 23;9(5):e97787. doi: 10.1371/journal.pone.0097787 (PMC4032256; doi:10.1371/journal.pone.0097787)
Supplement: Table S2 — HPV genome copy number in BC. HPV16 and 18 viral DNA in breast cancer (BC) and cervical cancer (CC) tissues were quantified by real-time quantitative polymerase chain reaction (PCR) using a TaqMan probes as mentioned in. Viral DNA was calculated based on the standard curve of control DNA. (DOC) [file pone.0097787.s006.doc]

**Table S2 HPV genome copy number in BC**.

| **Sample ID** | **HPV16** | **HPV16** | **HPV18** | **HPV18** | **% tumor cells in sample** |
| --- | --- | --- | --- | --- | --- |
|  | **DNA Chip** | **(copies/10^4^cell)** | **DNA Chip** | **(copies/10^4^cell)** |  |
| BC 284T | UD | 27 | UD | UD | 70 (DCIS) |
| BC 284N | + | 8 | UD | UD |  |
| BC 319T | + | 118 | UD | UD | 50 (IDC) |
| BC 391N | + | 34 | UD | UD |  |
| BC 420T | UD | UD | UD | UD | 50 (IDC) |
| BC 420N | + | 40 | + | 2 |  |
| BC 437T | UD | UD | UD | UD | 90 (IDC) |
| BC 437N | + | 58 | UD | UD |  |
| BC 441T | + | UD | UD | UD | 50 (IDC) |
| BC 441N | + | 28 | UD | UD |  |
| CC 053T | UD | UD | + | 31746 |  |
| CC 150T | + | 445466 | UD | UD |  |
| CC 128BN | + | 1100 | + | 4 |  |
| CC 352T | + | 18218 | UD | UD |  |
| T: Tumor sample , N: Non-tumor sample (adjacent to tumor), UD: undetectable | | | | | |
