# Supplementary material for: In Vivo and In Vitro Studies Suggest a Possible Involvement of HPV Infection in the Early Stage of Breast Carcinogenesis via APOBEC3B Induction
Source: PLoS One. 2014 May 23;9(5):e97787. doi: 10.1371/journal.pone.0097787 (PMC4032256; doi:10.1371/journal.pone.0097787)
Supplement: Text S1 — Supporting information text describes materials and methods for all of supporting figures and tables. (DOC) [file pone.0097787.s007.doc]

**Supporting Information Text**

**Materials and Methods**

**Quantification of viral DNA**

Viral DNA was quantified by real-time quantitative polymerase chain reaction (PCR) using a TaqMan Gene Expression Master Mix and 7500 Real-Time PCR system (Applied Biosystems) with following primers and TaqMan probe, as described previously^1,2^. Viral DNA was calculated based on the standard curve of control DNA.

HPV16 L1 (forward; 5’-TTGTTGGGGTAACCAACTATTTGTTACTGTT-3’, reverse; 5’-CCTCCCCATGTCTGAGGTACTCCTTAAAG-3’, probe; 6FMA-5’-GTCATTATGTGCTGCCATATCTACTTC-3’-TAMRA), HPV18 L1 (forward; 5’-GCATAATCAATTATTTGTTACTGTGGTAGATACCACT-3’, reverse; 5’-GCTATACTGCTTAAATTTGGTAGCATCATATTGC-3’, probe; 6FAM-5’-AACAATATGTGCTTCTACACAGTCTCCTGT-3’-TAMRA) and hGAPDH (forward; 5’-TGTGCTCCCACTCCTGATTTC-3’, reverse; 5’-CCTAGTCCCAGGGCTTTGATT-3’, probe; 6FAM-5’-AAAAGAGCTAGGAAGGACAGGCAACTTGGC-3’-TAMRA).

**Constructs**

HPV18-E6 and E7 genes were amplified using pSP-HPV18 plasmid gene specific primers as follows, E6: 5’-CGGAATTCGCCACCATGGCGCGCTTTGAGGATCC-3’ and 5’- ACCGCTCGAGTTATACTTGTGTTTCTCTGCGTCGTTG-3’, E7: 5’- CGGAATTCGCCACCATGCATGGACCTAAGGCAACATTG-3’, and 5’- ACCGCTCGAGTTACTGCTGGGATGCACACCAC-3’, and then cloned into pcDNA3.1 (+) (Lifetech) and pcDNA3.1-Flag vectors.

**Transfection**

HPV18 E6 or E7 plasmids were transfected into 293T and MCF10A cells with A3B-Pro and TK-Rluc as control. Cells were harvested at 48 hrs post-transfection followed by lysis. Luminescence was measured using Dula-Luciserase kit and Synergy H1 hybrid Multi-Mode Microplate reader, and HPV18 E7 and β-actin were detected using anti-Flag and β-actin Ab respectively.

HPV18 E6 or E7 specific-shRNA plasmids were transfected into A3B stably knocked down MCF10A-HPV18 cells followed by subjecting to western blot after lysis of cells. The γH2AX and β-actin were detected using anti- γH2AX and β-actin Ab.

**Reference**

1. Seaman WT, Andrews E, Couch M, Kojic EM, Cu-Uvin S, Palefsky J, et al. Detection and quantitation of HPV in genital and oral tissues and fluids by real time PCR. Virol J. 2010; **7**: 194.

2. Griscelli F, Barrois M, Chauvin S, Lastere S, Bellet D, Bourhis JH. Quantification of human cytomegalovirus DNA in bone marrow transplant recipients by real-time PCR. J Clin Microbiol. 2001; **39**(12): 4362-9.
